# Tangible co‐production? Engaging and creating with fathers

**DOI:** 10.1111/area.12691

**Published:** 2020-12-16

**Authors:** Iryna Culpin, Esther Dermott, Jonathan Ives, Julie MacLeavy

**Affiliations:** ^1^ Bristol Medical School University of Bristol Bristol UK; ^2^ School for Policy Studies University of Bristol Bristol UK; ^3^ Centre for Ethics in Medicine University of Bristol Bristol UK; ^4^ School of Geographical Sciences University of Bristol Bristol UK

**Keywords:** co‐production, creative methods, fathers, materiality, participatory research, tangible

## Abstract

This paper adds to an increasing body of social science literature, which engages with the research practice of “co‐production.” It aims to make a distinctive contribution by suggesting that what is produced under this process should be given greater attention. Previous literature has focused on the “co” (cooperative) element: debating whether and under what conditions wider participation between academic and non‐academic actors can be genuinely emancipatory, and the degree to which more radical research approaches centred on empowering marginalised groups have been usurped through management discourses of participatory governance. Drawing on a case study of a pilot project that developed support resources for new fathers under the auspices of a co‐production research design, the paper highlights the dynamics and limitations of the process, but additionally and distinctively suggests an important way in which the success of co‐production can be judged that includes practical and tangible outputs beyond academic knowledge and takes objects and materiality seriously as a dimension of co‐production in an academic setting.

## Graphical abstract

This paper draws on a case study of a pilot project that developed support resources for new fathers under the auspices of a co‐production research design. It suggests an important way in which the success of co‐production can be judged that includes practical and tangible outputs beyond academic knowledge and takes objects and materiality seriously as a dimension of co‐production in an academic setting.

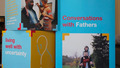

## 1 INTRODUCTION: CO‐PRODUCTION AS PARTICIPATION AND EMANCIPATION

Participatory research, at heart, challenges the idea of academic expertise as necessarily superior to the perspectives of those who are the subject of study, and maintains that better knowledge can be generated when different sources of “knowhow” are combined. The term “co‐production” implies a transdisciplinary collaboration between academics and external partners to co‐produce scholarly knowledge or outcomes of value. However, the term is used loosely within the social sciences to cover a range of related concepts and processes with a variety of implications for the final outcome. What unites co‐production projects is that they are undertaken with research users who are asked to take an active role in generating research findings or other outputs (Lunt et al., 2010). Co‐production as a term in public service delivery is related to participatory governance in that it suggests the active participation of citizens in capacity building and deliberative democracy. But while this approach can have an orientation to genuinely transform relationships (in the “towards utopia” envisaged by Bell & Pahl, 2016), it has also been viewed more suspiciously as a management tool that gives only the pretence of engagement and consultation (Kesby et al., 2007). It is difficult to design institutional arrangements that may engender successful co‐productive strategies by creating a sense of trust and empowerment among participants, and emerging forms of low or shallow citizen engagement may undermine alternative and more emancipatory practices (Mason, 2015). Governance actors are often envisaged in a coordinating role, working across and between different sectors and communities, such that “the mantra of co‐production serves, inadvertently, to re‐inscribe ‘business as usual,’ running the risk of co‐option and capture” of experiential expertise, locally generated innovation, and creativity that is grounded in specific contexts (Richardson et al., 2018, p. 146).

Within social science, co‐production as a research process is typically undertaken to secure a transfer of power from academics – who often come into research with specific questions and methodologies in mind – to research users by involving them in the design of research questions and the production of results based on lived experiences and profound understanding of “real life” challenges and needs. The justificatory narratives, then, are both ideological and practical, intended to democratise research (and improve ethical aspects such as consent) and enhance quality and relevance (Ives et al., 2013). The principle of engaging non‐academic participants in academic endeavour in a way in which their experience and voice are taken seriously has a long history. The emancipatory inclinations of feminist standpoint epistemology in the 1970s led to the insistence by political philosopher Nancy Hartsock and sociologist Dorothy Smith, among others, that research should be done not only “on” women but also “with” women and challenged the clear separation between the researcher and researched as subject and object (Hartsock, 1983; Smith, 1987, 1990). Similarly, critical race and post‐colonial work developed new forms of practice and theorising that challenged knowledge with its origins within white, Western settings (for example, Collins, 1990). Those involved in research with other marginalised groups have also adopted co‐production as part of a political project that seeks to empower those with relatively little authority in wider society. For instance, Victoria Murray argues that her research “seeks to blur the distinction between researcher and researched, allowing the voice of people with learning disabilities clearly to be heard” (2018, p. 424), drawing on the work of Chris Kiernan who proposes that “subjects” need to become co‐researchers (1999, p. 44; see also other contributions to the 1999 special section of *Area* 51(3), which focused on the geographies of co‐production, subtitled “learning from inclusive research approaches at the margins”). This lack of demarcation between academic and non‐academic participants is, then, intended not only to produce better research but, critically, to provide a route through which groups at the margins of decision‐making and control within societies can be given access to power.

While there are other academic traditions, largely outside of the social sciences, in which localised and “indigenous” knowledges play an important part – such as public history or anthropology (for a longer list of collaborative forms of research, see Facer & Enright, 2016) – we suggest that the dimensions of participation and emancipation are the two axes on which debate has most often centred. Specifically, discussions of the value of co‐production and the ability of academics to produce “genuine” co‐produced research have focused on the extent to which it is possible to combine different forms of expertise, agendas, and techniques to achieve this successfully (Bovaird & Loeffler, 2012): it is very challenging to reshape the relationships of politics and power in the research process and “facilitate interactions characterised by personal relations of solidarity and reciprocity … rather than individualised and instrumental relations” (Enright et al., 2016, p. 37). A similar tension between participatory use of shared resources, the “creative commons,” and political form of public access to collections in contemporary museum practice is captured by Heather Graham’s research on materiality and heritage (Graham, 2016, 2017). It is certainly not clear that co‐production is commonly – and unequivocally – achieved. It has been suggested that the dual aims of participation and emancipation will not always co‐exist. Forms of participation that are a requirement for impact agendas may offer only tokenistic recognition of values and preferences, as a remedy to demands from funders and the public for “relevance” (Dorling & Shaw, 2002), rather than benefitting communities. In other words, there is a sense that while high participation/high emancipation research should be the aim of engaging in co‐production, this is often not the outcome. The main points of this debate – what the “cooperation” element should look like and how this might be achieved – are of ongoing interest, but we suggest that a focus on this alone has led to a relative lack of engagement with the value of the *product* that is produced, and this also deserves attention. Put simply, in concentrating attention on *how* we co‐produce, *what* we co‐produce has been ignored.

The term co‐production has two parts that seek to locate ideas about engaging with different actors and creating outputs from their inputs (see also Alford, 2014). While we acknowledge that practice‐based research in arts disciplines has long produced objects as the output of research, geographical and social science research has tended to focus on the lived experience of co‐production, seeking to understand what induces different actors to participate (e.g., Warren, 2014). Turning to that which is produced as a consequence of engagement, this paper explores the value of the knowledge that is generated and how it is embodied within things – whether these are policy interventions, tangible objects, bodies, or other aspects of the physical world that individual actors inhabit.

Our case is a collaborative project involving four academics from different disciplines and a range of public actors (including Bluebell, a Bristol‐based non‐profit community organisation that supports families through depression related to pregnancy and birth; a local fathers group comprising a diverse group of men who were or soon to become fathers; and Blaise Castle House and Estate, a local museum and park). We focus on this project because it was explicitly designed to produce *tangible* outputs – directed by the funder whose focus was on funding “co‐creation” projects between academic researchers and the public to “make” something to help people “live well.” Our project “Conversations with Fathers” seemed to fit that brief (although it also challenged ideas about what co‐production is, or should be).

## 2 CONVERSATIONS WITH FATHERS

The Conversation with Fathers project was premised on the idea that becoming a parent is a life‐changing event, replete with uncertainties, and that there is a growing academic and practitioner focus on the need to support fathers as well as mothers (for example, Boyer et al., 2017a, 2017b; Halle et al., 2008). While fathers matter in their own right as well as being a significant source of support to mothers and children (Ives, 2019), they often lack formal and informal sources of support/guidance, arguably in part attributable to gendered/parenting norms (Aitken, 2009; Longhurst, 2017).

Previous research has highlighted that attempts to engineer support for fathers often tend to fail and are not taken up (e.g., Deave & Johnson, 2008). There may be a number of reasons for this. First, that those devising support tools for parenting (in relation to health, education, and employment settings) still tend not to prioritise the needs of fathers and therefore interventions, which replicate structures of informal or formal support for mothers, may not fit men. Second, where there have been institutional efforts to offer support there tends to be a focus on unidirectional information provision by “experts,” which focuses on the needs of children and pays little attention to what fathers themselves define as important, which may lead to their needs not being met. Third, affective/relational components of fatherhood tend to be under‐acknowledged in favour of a focus on practical aspects of childcare so that an important dimension of men’s parenting is not recognised within support mechanisms. Fourth, the dominant mode of communication with fathers is via written documents that require relatively high literacy skills and the ability and motivation to access them, meaning that interventions and support are likely to reach fathers who are already most active, visible, and engaged.

We reflected that an online resource, co‐produced with fathers, might help to address these challenges and would extend what is commonly included in everyday discourse about fatherhood. We envisaged a project that would take seriously from the outset the idea that men can find aspects of new fatherhood challenging and difficult. Through exploring collaboratively and collectively the heterogeneity of what is considered “normal” and “acceptable,” the aim was to recognise and acknowledge the nature and range of fathers’ feelings and practices in a way that could reduce the potential isolation and marginalisation of fathers. In addition, the project would strive to facilitate conversations around normal, everyday fatherhood that did not reproduce dichotomous discourse around either idealised or incompetent fathers often portrayed in popular culture. (As such, it had similarities to the work of Hanna (2018) and Tarrant and Neale (2017), which also took fathers’ voices seriously as a route to developing positive interventions.) The documented conversations would acknowledge that fatherhood is inherently relational – connected to and affected by a range of relationships – rather than defined through a narrow range of practical tasks. In capturing a wide spectrum of commentary and including diverse views and voices, the idea was that it would be possible to create a resource drawing on fathers’ personal accounts that could be valuable to a broader audience.

As a group of academics, we had a specific agenda, based on a particular view borne from our engagement with research on how fathers’ voices are, and fatherhood more generally is, often portrayed. We were also clear that creating a tangible resource, a “thing,” was central to the purpose of the project. Our view of a current lacuna in academic and support literature for fatherhood, and the associated rationale for a new kind of resource, was made explicit in the application for funding (which included, for example, time costed for the recording and editing of video material). This kind of narrative – which outlined our vision and its importance, the resources we needed and how those resources would be spent – is often essential to obtaining funding, and once funding was obtained we felt obliged to ensure that we followed that vision. For us, then, co‐production started with the idea of working with fathers to bring about a research‐driven vision – broadly to structure the nature and content of an online resource in a way that captured conversations (primarily using video) around fathering practices. We sensed an opportunity to bring different voices together to construct a resource, driven by fathers’ lived experience through collaboration and dialogue.

As a small project (less than £5,000 funding), the plan was to have an initial workshop with fathers to discuss and agree on areas that should be included in the resource – essentially what the conversations should be about. This would be followed by one‐on‐one filmed conversations capturing individual men’s reflections on fatherhood. The initial analysis and editing of these conversations would be conducted by the academics (primarily Jonathan Ives), who would then present this back to the group of fathers where it would form the basis of further discussion about how the film clips could be revised, developed, and used for an online resource, and what additional materials might be required. This plan exemplifies the co‐production mode of engagement insofar as it involved academics and users “working alongside each other at almost all stages” (Martin, 2010, p. 217). While it emerged in response to recent academic research highlighting the dilemmas associated with what living well as a father means in contemporary society, as well as the challenges of value pluralism when it comes to defining and practising “good fatherhood,” it aimed to co‐produce a resource to help and support men as fathers and to extend the repertoire of fathering practices to which men are exposed.

The initial workshop was arranged at a local community hall. This venue was chosen as children’s play activities were available (so that fathers could bring their children along) and because it was the location for a dads’ group (so for at least some of the men it was familiar and easy to access). Other contacts were also used to encourage attendance, including individuals who were fathers themselves or as gatekeepers to groups supporting dads and who some of the academics had been in contact with previously about their research. The result was a diverse group of fathers: men had children of different ages (from newborn to teenage); some of them were first‐time fathers, while others had experienced parenthood more than once, and there was one who was planning to become an adoptive father. There was a large variation in employment situations, and the group included unemployed, stay‐at‐home dads who were the primary care givers, shift workers, men who travelled long distances away from home, and self‐employed professionals. This meant there were differences in the incomes of these fathers and their households, as well as in the amount of time they could spend, and kinds of activities they did, with their children. There were also differences in their educational backgrounds. Some fathers were separated from partners, others were not, though as far as we could tell all either were in or had previously been in heterosexual relationships. Two of the fathers were from BAME groups, the rest were white.

The workshop conversation, after introductions that emphasised the range of backgrounds outlined above and saw us describing the broad aims of the project, began with an open question about what fathers needed in terms of content and format. While our intention was always to provide some online form of support/advice/information, we wanted to sense‐check our prioritisation of this form of output with the fathers as well as discuss the content. At this point it became evident that, perhaps unsurprisingly in retrospect, the diverse set of fathers had an equally diverse set of views on what they would have liked in the past, and what would be valuable for them now. Some of the differences reflected the ages of their children and (re‐)emphasised that parenting varies immensely depending on whether a child is a newborn, a toddler, or a teenager, and also reflected the significance of other social characteristics. While some of the fathers were enthusiastic about the accessibility and value of an online resource, others made it clear that with limited access and ability in relation to computers they would never make use of it. Some fathers felt that there was a real value in bringing groups of dads together to share information and experiences and that an online resource could facilitate this in a way that was difficult to achieve in other ways across large distances and in relation to work commitments, while others expressed a strong view that face‐to‐face opportunities for sharing were preferable. When it came to discussing content, there was a degree of consensus that knowing of the existence of other fathers in similar circumstances to their own could be helpful, but the men had divergent views about what they wanted the content of the conversations to focus on; for example, childcare tips including how to deal with crying and feeding; managing/negotiating household roles; developmental stages; discipline and behaviour management; work–life balance; and partner relationships.

This level of variation was helpful in that it confirmed our view about the importance of reflecting heterogeneity in fatherhood and indeed that the practices of fathering exist in all social contexts. But it was also problematic, insofar as it became clear that the kind of group co‐production we had in mind would be next to impossible: first, there was a push away from the type of output we were already committed to, and second, there was a lack of consensus about what the conversations should focus on. It highlighted things that we might have done differently in the lead up to the project (such as focusing on a particular sub‐group of fathers or being more overtly prescriptive about the resource that would be developed) so that a consensus view was more likely to emerge. It also drew attention to the way we often assume that consensus will emerge if we simply use the “right” research design, whereas researchers may need to engage more with the reality of dissensus and genuine plurality to develop deliberative successful and just interventions (as discussed by Mouffe (2013) in relation to the political sphere). Practically, the next steps did not seem obvious. The project and funds were time limited, and we were committed to producing some kind of tangible output. We compromised by piloting and testing various ways to engage in conversations with fathers and about fathers, aiming to produce a range of differently presented conversation material which we could then, with future funding, evaluate further and use as the basis for a more robust co‐production project. However, we did prioritise our agenda in that we did not ask the men, either individually or collectively, what to do next.

We undertook four connected activities. (1) We filmed a series of one‐on‐one conversations with fathers who had a particular experience they wanted to talk about. For this we partnered with Bluebell, and also asked men from our initial group of fathers if they would like to participate. Two men were recruited through Bluebell, and one through the fathers’ group. (2) We constructed a (temporary) video booth where men could go and speak to a camera, alone, about any aspect of fatherhood they wanted. This was done at a public event set up at the University of Bristol to showcase the range of projects the Brigstow Institute was funding. All of the projects at this event had a participatory element, and the event was attended by members of the public and university staff and students. (3) At the same event we set up a “conversation wall” where fathers could write about either their own experiences of fatherhood, or their own fathers, and pin them up for others to read. (4) During a family day at Blaise Castle in Bristol we talked to visitors about the project and specifically invited men to write down their thoughts about fatherhood and/or their own fathers, or speak to camera. The event was trailed in advance on local BBC radio, and a local magazine (Bristol 24/7) reported on it.

All of these activities aimed to involve fathers front and centre, but one transformative realisation that we came to through trialling them was that our initial framing of the project was problematic. We had begun anticipating having conversations with fathers, but as the various public activities took place it became increasingly obvious that there was strong appetite among people who were not fathers to have conversations about fatherhood – reflecting the fact that everyone has an interest in fatherhood – one way or another. In addition to fathers talking about fatherhood, adult children asked to write notes to or about their own fathers – living and deceased – saying things they have not said or felt they could not say previously. Partners of fathers engaged in conversations (written and verbal) about their own fathers or the fathers of their children. Some of these conversations were formal and were then recorded on video, some were recorded on paper, and some were not recorded at all. Some conversations were had in public, some in private, and two were subsequently broadcast (over the radio and local online news). As such, these public activities seemed to evolve into an unanticipated form of co‐production, which transformed the project into something far broader than we had originally intended, but also kept it well within the remit that we felt we had to stay within (given what we had promised when applying for funding).

We ended up having lots of different conversations with and about fathers, with people we had not set out to speak to, and this change was an unplanned evolution brought about simply by doing what we set out to do – having and recording conversations with fathers – but then allowing people to define what that meant for themselves. “Conversations with fathers” became “having conversations about fatherhood” (less catchy, but perhaps more authentic) and included: fathers talking to fathers; partners talking to the fathers of their children; children talking to and about their fathers; reporters, broadcasters, and academics talking to the public about fatherhood; and the public talking to us about fathers. In a sense, the conversations generated through these activities, and the process of publicly thinking and talking about fatherhood, became the output – the intangible product – that arose while trying to create something tangible.

Off the back of those intangible outputs, we were also able to create something tangible, in the form of a website that acts as hub for two kinds of output (http://www.bristol.ac.uk/conversations‐with‐fathers/). The first is two “conversations with fathers” videos, featuring Louis and Matt talking about their negative and positive experiences of fatherhood – covering mental health, erectile dysfunction, and spousal relationships. (Another video, with Mark talking about raising daughters and adoption, is in production.) These videos were filmed and produced by a local videographer (Ed Barnes Videography), who is a father himself and was present at all of our events. The raw film was transcribed and then edited into 12 thematic clips (by Jonathan Ives) so that the “conversations” were more easily navigable and short enough to be easily consumed. The natural ebb and flow of a long conversation meant that topics were not always covered in sequence, and topics discussed earlier were often returned to later. This made it necessary to edits bits of the conversation together to produce coherent clips, and narrative and thematic content were privileged over preserving the timeline and structure of the original conversations. Once edited, clips were sent to the fathers involved, who were given the opportunity to ask for changes; both gave consent for publication with no amendments. The 12 clips are currently hosted on YouTube. The second output is an online version of the “conversations wall,” where we have displayed selected messages. Many of most powerful messages were from children to their own fathers, and so we called this section “To fathers, from children.” There is a contact page where we invite people to send in their own messages.

## 3 ASSESSING “CONVERSATIONS WITH FATHERS” – PRODUCT OR PROCESS?

The nature of our project implied a series of short‐term partnerships with different men, with the potential to empower fathers to share their experiences and enhance their involvement in both public and private conversations about parenthood and childrearing. This participatory approach facilitated a more balanced researcher–research user relationship during the project design and participation stages, but in other ways it fell short of the “co‐production ideal” we envisaged.

We attempted to approach our project as an ongoing collaboration between researchers and men who are fathers from outside the academic environment, with the aim of creating a specific kind of product. However, the variety and breadth of men’s voices, and the range of experiences that our conversations encompassed, somewhat conditioned the narrative that could be constructed for the purposes of creating a tangible support resource. The power dynamics inherit in assembling a single narrative from the diverse, often conflicting, needs of the fathers presented challenges that framed and re‐framed the nature of our project. Focusing on the process allows the description of procedural value, by which we mean the project can be assessed on the extent to which it follows a specified process (or procedure). As such, if we focus on the process, from conception through to completion, our project might be considered to have failed, whereas other projects of this kind succeed because they “fit” with an accepted model of co‐production.

It is a given that co‐produced research is complex, and that some mess and “failure” are inevitable. Our sense of failure and mess stems from the lack of consensus and our inability to maintain engagement with fathers as co‐producers throughout the project. Note that this paper is not co‐written with fathers (at the time of writing it made little sense to go back to invite them as co‐authors to report on activities in which they were not fully involved). But these failures precipitate a reflection on what success looks like and what the value of consensus is. Just as dissensus may be inevitable, consensus itself does not necessarily confer justification and need not define success. It is not necessarily a problem if research follows a broad agenda set by researchers, so long as that agenda can be justified. It is only a problem if disingenuously presented. The co‐production we ended up engaging in was perhaps a hybrid form, with the goals determined and justified by us as researchers, and the products/outputs shaped to varying degrees with different publics.

The project did lead to a series of differently co‐produced outputs – some more or less tangible than others – that seem to have value as co‐created “things” and so succeeded in that domain. However, beyond the fact of their existence, assessing the value of these products is difficult. When we focus on the product, assessing value is challenging because there are so many ways that a product can have value, or be valued. We could calculate how many times video clips have been accessed (a relatively small number in the realm of social media) but we cannot know whether they benefitted the people who watched them, or in what way. Similarly, we cannot measure the value of the conversations we had at our public events, or the catharsis a person might find from writing a message on the conversation wall, or from reading a message that resonates with them. The challenges of knowing the intangible value of tangible objects are twofold. First is the challenge of determining what measure of success to use and how to measure it. Access figures and qualitative feedback seem themselves to be of limited value in really capturing reach, engagement, and resonance. Second, even this type of measurement requires sustained engagement from both users and developers. The product (e.g., the website) needs to be maintained and monitored, and this is resource intensive. To justify resource for maintenance it is necessary to demonstrate value and impact – and so there is a chicken and egg problem that needs resolving – and this is going to be a potential problem for any co‐production research that is directed at product rather than process.

## 4 CONCLUSION

The co‐production of a web resource to help men “live well” as fathers constitutes an interesting example of interaction between academics and research users because it demonstrates that the engaged scholarship that is now so heavily promoted is not always possible even when it seems plausible at the outset. There is the potential for co‐production to deliver a shift in the way knowledge and research is produced, giving voice and prominence to different communities of interest. Our example, however, highlights how this relies on a degree of consensus between the non‐academic partners who are involved, as well as between academic researchers and other users. We suggest that dissensus may be more typical than is evident from most published accounts of research and that taking this lack of agreement seriously and thinking about how to capture and work with plural views should have greater prominence.

What stands out in our review of this project is that the value of producing a physical resource – a tangible output beyond concepts, or ideas for thinking about fatherhood, or a transformation in men’s own orientations – is also potentially of value and should be brought into academic reflections on the potential value of co‐production. For some practice‐based disciplines, say engineering or medicine, the formulation of an academic project that produces a material object that can be of benefit to someone in society is hardly revolutionary outcome but is intrinsic to valuable, problem‐solving research. In geography and the social sciences, it is less usual to focus on a material object in this way: research intervention or impact is often measured in terms of policy briefings or proposals for practitioners to engage differently with relevant groups. This paper proposes that this form of explicitly practice‐based, problem‐oriented research may be a fruitful development, notwithstanding the outcome of this specific project.

## Data Availability

Data sharing not applicable to this paper as no datasets were generated or analysed during the current study.

## References

[area12691-bib-0001] Aitken, S. (2009). The awkward spaces of fathering. Farnham, UK: Ashgate.

[area12691-bib-0002] Alford, J. (2014). The multiple facets of co‐production: Building on the work of Elinor Ostrom. Public Management Review, 16, 299–316. 10.1080/14719037.2013.806578

[area12691-bib-0003] Bell, D. M. , & Pahl, K. (2016). Co‐production: towards a utopian approach. International Journal of Social Research Methodology, 21, 105–117. 10.1080/13645579.2017.1348581

[area12691-bib-0004] Bovaird, T. , & Loeffler, E. (2012). From engagement to co‐production: The contribution of users and communities to outcomes and public value. Voluntas: International Journal of Voluntary and Nonprofit Organizations, 23, 1119–1138. 10.1007/s11266-012-9309-6

[area12691-bib-0005] Boyer, K. , Dermott, E. , James, A. , & MacLeavy, J. (2017a). Regendering care in the aftermath of recession? Dialogues in Human Geography, 7, 56–73. 10.1177/2043820617691632

[area12691-bib-0006] Boyer, K. , Dermott, E. , James, A. , & MacLeavy, J. (2017b). Men at work? Debating shifting gender divisions of care. Dialogues in Human Geography, 7, 92–98. 10.1177/2043820617691630

[area12691-bib-0007] Deave, T. , & Johnson, D. (2008). The transition to parenthood: What does it mean for fathers? Journal of Advanced Nursing, 63, 626–633. 10.1111/j.1365-2648.2008.04748.x 18808584

[area12691-bib-0008] Dorling, D. , & Shaw, M. (2002). Geographies of the agenda: Public policy, the discipline and its (re)‘turns’. Progress in Human Geography, 26, 629–641. 10.1191/0309132502ph390oa

[area12691-bib-0009] Enright, B. , Facer, K. , & Larner, W. (2016). Reframing co‐production: Gender, relational academic labour and the university. In E. Jupp , J. Pykett , & F. M. Smith (Eds.), Emotional States: Sites and spaces of affective governance (pp. 36–52). Oxford, UK: Taylor and Francis.

[area12691-bib-0010] Facer, K. , & Enright, B. (2016). Creating Living Knowledge: The Connected Communities Programme, community‐university partnerships and the participatory turn in the production of knowledge. Bristol, UK: Arts and Humanities Research Council.

[area12691-bib-0011] Graham, H. C. (2016). The ‘co’ in co‐production: Museums, community participation and Science and Technology Studies. Science Museum Group Journal, 5, 5. 10.15180/160502

[area12691-bib-0012] Graham, H.C. (2017). Publics and commons: The problem of inclusion for participation. ARKEN Bulletin, 7, 150–167.

[area12691-bib-0013] Halle, C. , Dowd, T. , Fowler, C. , Rissel, K. , Hennessy, K. , MacNevin, R. , & Nelson, M. A. (2008). Supporting fathers in the transition to parenthood. Contemporary Nurse, 31, 57–70. 10.5172/conu.673.31.1.57 19117501

[area12691-bib-0014] Hanna, E. (2018). Supporting young men as fathers: Gendered understandings of community group‐based provision. Basingstoke, UK: Palgrave MacMillan.

[area12691-bib-0015] Hartsock, N. (1983). The feminist standpoint: Developing the ground for a specific feminist historical materialism. In S. Harding , & M. Hintikka (Eds.), Discovering reality: Feminist perspectives on epistemology, metaphysics, methodology and the philosophy of science. Dordrecht, Netherlands: Reidel Publishing.

[area12691-bib-0016] Collins, P.H. (1990). Black feminist thought: Knowledge, consciousness and the politics of empowerment. Boston, MA: Unwin Hyman.

[area12691-bib-0017] Ives, J. (2019). Framing fatherhood: The ethics and philosophy of researching fatherhoods. In E. Dermott , & C. Gatrell (Eds.), Fathers, families and relationships: Researching everyday lives. Bristol, UK: Bristol University Press.

[area12691-bib-0018] Ives, J. , Damery, S. , & Redwood, S. (2013). PPI, paradoxes and Plato: Who's sailing the ship? Journal of Medical Ethics, 39, 186–187. 10.1136/medethics-2011-100150 22267385

[area12691-bib-0019] Kesby, M. , Kindon, S. , & Pain, R. (2007). Participation as a form of Power. In S. Kindon , R. Pain , & M. Kesby (Eds.), Participatory action research approaches and methods: Connecting people, participation and place (pp. 19–25). Abingdon, UK: Routledge.

[area12691-bib-0020] Kiernan, C. (1999). Participation in research by people with learning disability: origins and issues. British Journal of Learning Disabilities, 27, 43–47. 10.1111/j.1468-3156.1999.tb00084.x

[area12691-bib-0021] Longhurst, R. (2017). Reflecting on regendering care at different scales: Bodies, home, community and nation. Dialogues in Human Geography, 7, 79–82. 10.1177/2043820617691598

[area12691-bib-0022] Lunt, N. , Shaw, I. , & Fouché, C. (2010). Practitioner research: collaboration and knowledge production. Public Money and Management, 30, 235–242. 10.1080/09540962.2010.492185

[area12691-bib-0023] Martin, S. (2010). Co‐production of social research: strategies for engaged scholarship. Public Money and Management, 30, 211–218. 10.1080/09540962.2010.492180

[area12691-bib-0024] Mason, K. (2015). Participatory action research: coproduction, governance and care. Geography Compass, 9, 497–507. 10.1111/gec3.12227

[area12691-bib-0025] Mouffe, C. (2013). Agonists: Thinking the world politically. London, UK: Verso.

[area12691-bib-0026] Murray, V. (2018). Co‐producing knowledge: Reflections on research on the residential geographies of learning disability. Area, 51, 423–432. 10.1111/area.12491

[area12691-bib-0028] Richardson, L. , Durose, C. , & Perry, B. (2018). Coproducing urban governance. Politics and Governance, 6, 145–149. 10.17645/pag.v6i1.1485

[area12691-bib-0029] Smith, D. (1987). The everyday world as problematic: A feminist sociology. Boston, MA: Northeastern University Press.

[area12691-bib-0030] Smith, D. (1990). The conceptual practices of power: A Feminist sociology of knowledge. Boston, MA: Northeastern University Press.

[area12691-bib-0031] A. Tarrant , & B. Neale (Eds.) (2017). Learning to Support Young Dads: Responding to Young Fathers in a Different Way: Project Report. Retrieved from https://dratarrant.files.wordpress.com/2017/04/syd‐report‐final‐19‐4.pdf

[area12691-bib-0032] Warren, S. (2014). ‘I want this place to thrive’: Volunteering, co‐production and creative labour. Area, 46, 278–284. 10.1111/area.12112

